# eIF4E Phosphorylation Influences *Bdnf* mRNA Translation in Mouse Dorsal Root Ganglion Neurons

**DOI:** 10.3389/fncel.2018.00029

**Published:** 2018-02-06

**Authors:** Jamie K. Moy, Arkady Khoutorsky, Marina N. Asiedu, Gregory Dussor, Theodore J. Price

**Affiliations:** ^1^School of Behavioral and Brain Sciences, University of Texas at Dallas, Richardson, TX, United States; ^2^Department of Pharmacology, University of Arizona, Tucson, AZ, United States; ^3^Department of Anesthesia, McGill University, Montréal, QC, Canada; ^4^Alan Edwards Centre for Research on Pain, McGill University, Montréal, QC, Canada

**Keywords:** eIF4E phosphorylation, BDNF, DRG, pain, hyperalgesic priming

## Abstract

Plasticity in dorsal root ganglion (DRG) neurons that promotes pain requires activity-dependent mRNA translation. Protein synthesis inhibitors block the ability of many pain-promoting molecules to enhance excitability in DRG neurons and attenuate behavioral signs of pain plasticity. In line with this, we have recently shown that phosphorylation of the 5′ cap-binding protein, eIF4E, plays a pivotal role in plasticity of DRG nociceptors in models of hyperalgesic priming. However, mRNA targets of eIF4E phosphorylation have not been elucidated in the DRG. Brain-derived neurotrophic factor (BDNF) signaling from nociceptors in the DRG to spinal dorsal horn neurons is an important mediator of hyperalgesic priming. Regulatory mechanisms that promote pain plasticity via controlling BDNF expression that is involved in promoting pain plasticity have not been identified. We show that phosphorylation of eIF4E is paramount for *Bdnf* mRNA translation in the DRG. *Bdnf* mRNA translation is reduced in mice lacking eIF4E phosphorylation (*eIF4E^S209A^*) and pro-nociceptive factors fail to increase BDNF protein levels in the DRGs of these mice despite robust upregulation of *Bdnf-201* mRNA levels. Importantly, bypassing the DRG by giving intrathecal injection of BDNF in *eIF4E^S209A^* mice creates a strong hyperalgesic priming response that is normally absent or reduced in these mice. We conclude that eIF4E phosphorylation-mediated translational control of BDNF expression is a key mechanism for nociceptor plasticity leading to hyperalgesic priming.

## Introduction

Translational control of gene expression is a key process for the regulation of plasticity in the nervous system. Multiple lines of evidence indicate that translation control plays a critical role in pathological pain plasticity (Khoutorsky and Price, 2017). In the peripheral nervous system (PNS), injured dorsal root ganglion (DRG) neurons undergo changes that shift the excitability profile of the nociceptor (Price and Gold, 2017). In the DRG, pain-inducing ligands act via their receptors to activate two major kinase pathways, mechanistic target of rapamycin (mTOR) and mitogen-activated protein kinase (MAPK). These pathways converge on the 5′ cap of mRNAs to initiate protein synthesis via the eukaryotic translation initiation factor (eIF) 4F complex formation (Melemedjian et al., 2010). This complex is comprised of three proteins: the scaffolding protein eIF4G, the RNA helicase eIF4A, and the 5′ cap-binding protein eIF4E. Activated mTOR phosphorylates 4E-binding proteins (4E-BPs), releasing eIF4E from their inhibition and thereby promoting eIF4F complex formation (Sonenberg and Hinnebusch, 2009). Moreover, activated MAPKs stimulate eIF4E phosphorylation at serine 209 through MAPK interacting kinases (MNKs) 1/2 (Pyronnet et al., 1999; Waskiewicz et al., 1999). We have recently shown that phosphorylation of eIF4E (p-eIF4E) plays a critical role in the development of nociceptive plasticity and hyperalgesic priming (Moy et al., 2017). Specific mRNA targets of eIF4E phosphorylation in the DRG have not been elucidated.

Previous studies have shown that the mRNA 5′ untranslated region (5′ UTRs) has a strong influence on translation efficiency and signaling pathways that regulate translation of specific mRNAs. For example, mRNAs containing 5′ UTR terminal oligopyrimidine sequences or several GG pairs in a short nucleotide sequences called G-quadruplexes rely on mTOR activation (Thoreen et al., 2012) or eIF4A helicase activity (Wolfe et al., 2014), respectively. Additionally, mRNAs with CERT domains rely on eIF4E availability for their translation (Truitt et al., 2015). It is clear that eIF4E phosphorylation regulates the translation of a subset of mRNAs but the factors that control this specificity have not been identified (Furic et al., 2010; Herdy et al., 2012). Having said that, several individual mRNA targets of eIF4E phosphorylation have been identified in mouse embryonic fibroblasts derived from *eIF4E^S209A^* mice (Furic et al., 2010). These mice have normal levels of total eIF4E, but the protein is unphosphorylated (Furic et al., 2010; Cao et al., 2015; Moy et al., 2017). Additionally, matrix metalloproteinases (MMPs) 2 and 9 mRNAs have been shown to be regulated by eIF4E phosphorylation in the central nervous system (CNS) (Gkogkas et al., 2014). Targets of eIF4E phosphorylation in the DRG have not been identified.

Brain-derived neurotrophic factor (BDNF) is a well-known mediator of pain plasticity and is released by a subset of DRG neurons to act on postsynaptic (Zhao et al., 2006; Zhou et al., 2008; Melemedjian et al., 2013), and potentially presynaptic (Chen et al., 2014) tyrosine receptor kinase type B (trkB) in the dorsal horn. BDNF is a plasticity-related neurotrophin that is critical for induction and maintenance of long-term potentiation (LTP) in the brain and dorsal horn of the spinal cord (Lu et al., 2008). Interestingly, BDNF application to cortical neurons stimulates eIF4E phosphorylation through MNK1 (Panja et al., 2014; Genheden et al., 2015). *Bdnf* mRNA and protein expression are increased in DRG nociceptors following NGF exposure or inflammatory injury (Kerr et al., 1999; Mannion et al., 1999). We have previously shown that BDNF signaling is required for the generation of hyperalgesic priming (Melemedjian et al., 2013, 2014), which led us to hypothesize that eIF4E phosphorylation, which also plays a key role in hyperalgesic priming (Moy et al., 2017), may regulate *Bdnf* mRNA translation. We identify that the *Bdnf-201* mRNA isoform as a *bona fide* eIF4E phosphorylation translation target in the DRG providing evidence that this signaling pathway is engaged in driving phenotypic changes in BDNF protein expression that generate persistent pain plasticity.

## Materials and Methods

### Animals

All mice were bred and housed in a 12-h/12-h light/dark cycle starting at 7AM. Mice were housed with food and water available *ad libitum. eIF4E^S209A^* mice on a C57BL/6 background were gifted to us from the Sonenberg laboratory at McGill University (Furic et al., 2010), and bred at The University of Arizona or The University of Texas at Dallas to produce experimental animals. *Bdnf*^+/-^ mice were obtained from The Jackson Laboratories (strain B6.129S4-*Bdnf*^tm1Jae^/J). All mice weighed approximately 20–25 g prior to experimental use. Genotypes of the mice were determined by polymerase chain reaction (PCR) through DNA extraction of ear clips at 3–4 weeks old. The Institutional Animal Care and Use Committees at The University of Arizona, The University of Texas at Dallas, or McGill University approved all use of animal procedures. Procedures were performed according to the guidelines provided by the International Association for the Study of Pain.

### Behavior

Both male and female mice were used for our behavioral studies (WT: 4 males, 2 females; *eIF4E^S209A^*: 5 males, 3 females). Testing was performed during the hours of 9AM and 4PM. Mice were habituated in their testing chambers for approximately 1 h prior to beginning the experiment. Hindpaw mechanical thresholds were determined by using the up-down method as described in Chaplan et al. (1994) using calibrated von Frey filaments (Stoelting Company, Wood Dale, IL, United States). BDNF intrathecal injections were administered in a 5 μL volume via a 3012-gauge needle (Hylden and Wilcox, 1980). The experimenter (*MNA)* was blinded to the genotype of the mice.

### Western Blotting

Male mice were anesthetized with ketamine and perfused with ice-cold 1× phosphate-buffered saline (PBS) solution to flush out the blood. Tissues were then isolated and flash frozen via dry ice. Frozen tissues were placed in ice cold lysis buffer (50 mM Tris pH 7.4, 150 mM NaCl, 1 mM EDTA pH 8.0, and 1% Triton X-100) containing protease and phosphatase inhibitors cocktails (Sigma–Aldrich) and homogenized using a pestle or sonication. Samples were centrifuged at 14,000 rpm for 15 min at 4°C and the supernatant containing protein extracts was collected. Protein concentrations were assessed using the Pierce BCA protein assay kit (ThermoFisher Scientific) as directed. A total of 10–15 μg of protein was mixed with Laemmli sample buffer (Bio-Rad) and 2-mercaptoethanol and was heated at 95°C for 5 min. Samples were loaded into each well of a 10% SDS–PAGE gel along with 15 μL of Precision plus protein kaleidoscope prestained protein standards (Bio-Rad). Proteins were transferred to a 0.45 PVDF membrane (Millipore, Billierca, MA, United States) at 30 V overnight or 85 V for 1 h at 4°C. Membranes were blocked using 5% non-fat dry milk in 1× Tris Buffer Saline-Tween (TTBS) prior to primary antibody incubation. Bands were visualized using film (Kodak) or with a Bio-Rad ChemiDoc Touch. Overexposed or saturated pixels detected by the ChemiDoc Touch were excluded from analysis. Analysis was performed using ImageJ version 1.48 or Image Lab version 6.0.

### Antibodies and Chemicals

The BDNF antibodies were purchased from Developmental Studies Hybridoma Bank at the University of Iowa (mouse #9; Iowa City, IA, United States) and Sigma–Aldrich (rabbit; St. Louis, MO, United States). Phospho-eIF4E, GAPDH, and trkB antibodies were obtained from Cell Signaling Technology (Danvers, MA, United States). PAR2 agonist, 2-aminothiazol-4-yl-LIGRL-NH_2_ (2at-LIGRL), was synthesized as described previously (Boitano et al., 2011). Human recombinant BDNF was purchased from R&D Systems (Minneapolis, MN, United States). Prostaglandin E_2_ (PGE_2_) was purchased from Cayman chemicals (Ann Arbor, MI, United States). All other chemicals were attained from ThermoFisher Scientific (Waltham, MA, United States).

### Quantitative Reverse Transcriptase – Polymerase Chain Reaction (qRT-PCR)

**FIGURE 1:**
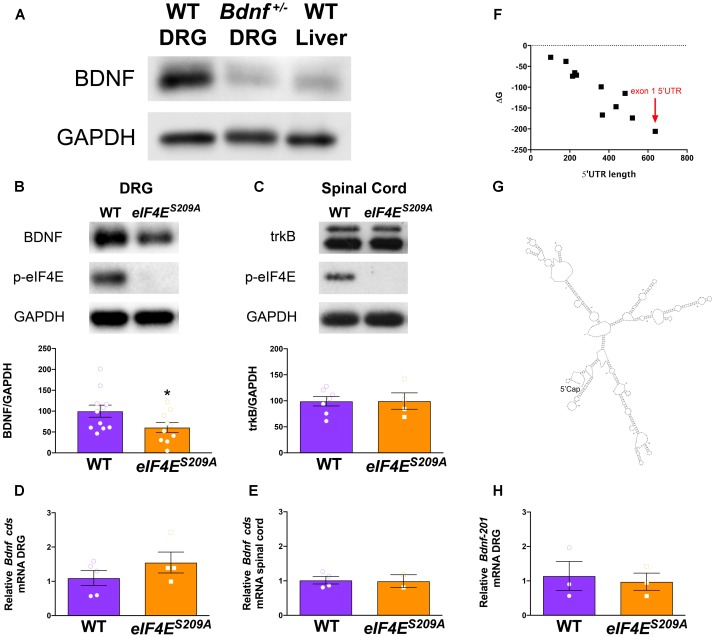
Decreased *Bdnf* mRNA translation in *eIF4E^S209A^* mouse DRG. BDNF antibody was verified by immunoblotting against *Bdnf*^+/-^ DRGs and WT liver showing reduced levels of BDNF protein compared to WT DRGs **(A)**. *eIF4E^S209A^* mouse DRGs **(B**, *n* ≥ 4, *t* = 3.238, *df* = 7, ^∗^*p* = 0.0143, *t*-test) showed lower levels of BDNF protein expression compared to WT (*n* ≥ 4, ^∗^*p* < 0.05, *t*-test) but equal levels of trkB expression **(C)** in the spinal cord (*n* ≥ 5, *t*-test). **(D,F)** While BDNF protein levels were lower in *eIF4E^S209A^* DRG compared to WT DRG, total-*Bdnf* mRNA levels were equal in DRG **(D)** and in spinal cord **(E**, *n* ≥ 4, *t*-test). **(F)** Delta G (ΔG) free energy map of *Bdnf* transcript variants plotted by 5′ UTR length. *Bdnf-201* transcript is shown by the red arrow. **(G)** Structure of *Bdnf exon 1* 5′ UTR as predicted by mfold: http://unafold.rna.albany.edu/?q=mfold. **(H)** *Bdnf-201* mRNA expression was equal in DRG between *eIF4E^S209A^* and WT DRG (*n* ≥ 4, *t*-test).

Lumbar DRGs and spinal cords were isolated from 3 to 6 male mice per genotype and flash-frozen on dry ice and stored at -80°C until ready to be processed. Tissues were homogenized using a pestle and total RNA was extracted using RNAqueous Total RNA Isolation kits (ThermoFisher Scientific). RNA was subsequently treated with TURBO DNase (ThermoFisher Scientific) according to the manufacturer’s instructions. RNA concentration was measured on a NanoDrop 2000 (ThermoFisher Scientific). cDNA was synthesized using iScript Reverse Transcriptase (Bio-Rad). qRT-PCR was done using a Applied Biosystems Lightcycler 7500 real-time PCR system using iTaq Universal SYBR Green Supermix (Bio-Rad) according to the manufacturer’s instructions with three technical replicates per biological replicate (averages of the technical replicates per biological replicate are reported) using primers pairs: *Gapdh* forward 5′-TGACCTCAACTACATGGTCTACA-3′ and *Gapdh* reverse 5′-CTTCCCATTCTCGGCCTT G-3′, *Bdnf cds* forward 5′-GCGGCAGATAAAAAGACTGC-3′ and *Bdnf cds* reverse 5′-GCAGCCTTCCTTGGTGTAAC-3′, and *Bdnf-201* forward 5′-TGTTGGGGAGACAAGATTTT-3′ and *Bdnf-201* reverse 5′-CGTGGACGTTTACTTCTTTC-3′. *Bdnf* primers were the same as in Matsuoka et al. (2007). Primers were made by Integrated DNA Technologies (Coralville, IA, United States). Data were analyzed as 2^-ΔΔ*C*_T_^ and normalized as shown in the Section “Results.” Experiments using this method of qRT-PCR (**Figures 1D,E,H**, **3C–F** and **Supplementary Figure S3** were performed at The University of Texas at Dallas.

### Polysome Profile Analysis

**FIGURE 2:**
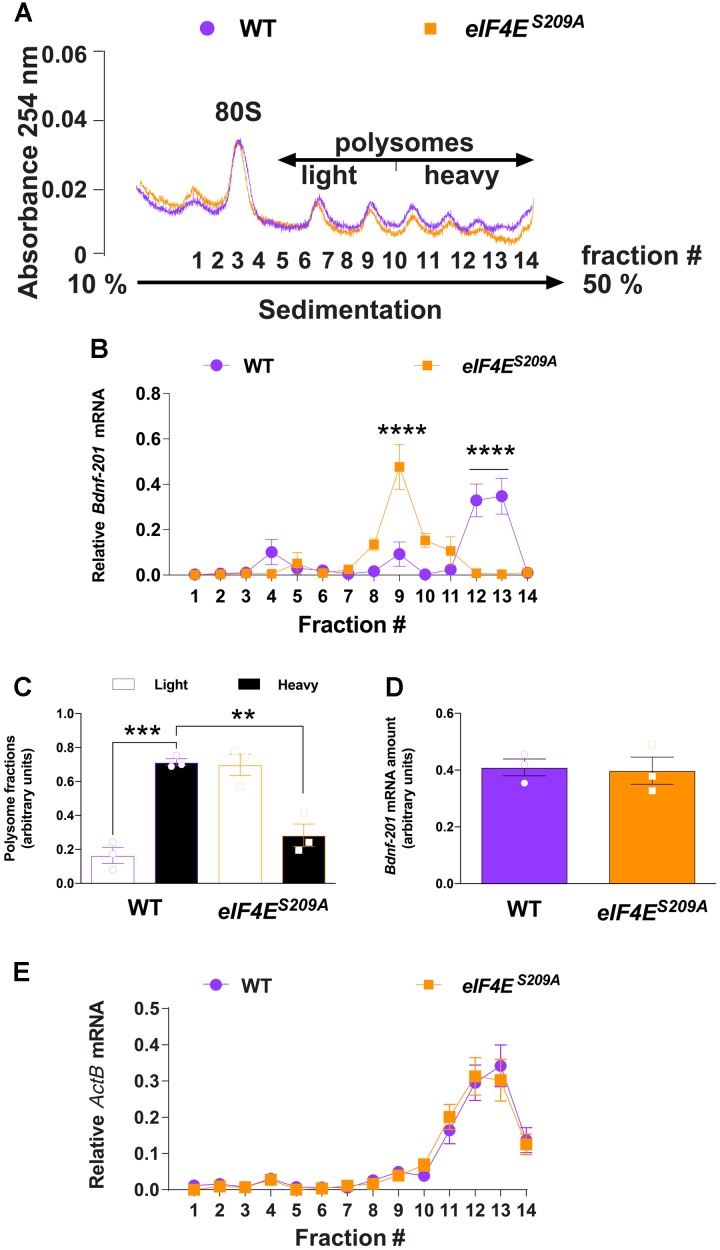
*Bdnf-201* mRNA localizes to lighter polysomes in *eIF4E^S209A^* mouse DRGs. Polysome profiling of DRG lysates from WT and *eIF4E*^S209A^ mice **(A)**. **(B,C)** The relative amount of *Bdnf-201* mRNA in the heavy (fraction 10–14) vs. light (fraction 5–9) polysome fractions was decreased in *eIF4E^S209A^* mouse DRG indicating decreased *Bdnf-201* mRNA translation [**B**, *n* = 3, *F*(13,56) = 12.5, *p* < 0.0001; *post hoc* Bonferroni’s *^∗∗∗∗^p* < 0.0001; **C**, *n* = 3, *F*(3,8) = 28.78, *p* = 0.0001; *post hoc* Bonferroni’s ^∗∗∗^*p* = 0.0002, ^∗∗^*p* = 0.0011 one-way ANOVA]. **(D)** Total *Bdnf-201* mRNA amount did not differ between genotypes in samples used for polysome analysis (*n* = 3). **(E)** *ActB* mRNAs localize to heavy polysomes in both WT and *eIF4E^S209A^* DRGs.

Lumbar and thoracic DRGs were isolated from 5 mice per genotype and flash-frozen on dry ice. The DRGs were placed in chilled lysis buffer containing: 40 mM Tris–HCl, pH 7.4, 150 mM NaCl, 5 mM MgCl_2_, 100 μg/ml cycloheximide, 1 mM DTT, 8% glycerol, and RNase inhibitors (RNAsin, Promega, Madison, WI, United States), and the tissue was subjected to brief homogenization using a glass homogenizer. The homogenized material was spun at 16,000 relative centrifugal force (RCF) for 10 min at 4°C, and the supernatant was loaded on a 10–50% w/w sucrose gradient in 40 mM Tris–HCl, pH 7.4, 150 mM NaCl, 5 mM MgCl_2,_ 100 μg/ml cyclohexamide, and RNAsin, and centrifuged at 36,000 RPM for 2.5 h at 4°C in Optima L-80 XP ultracentrifuge (Beckman Coulter, Pasadena, CA, United States) using an SW40 rotor. Polysome analysis was performed by measuring the optical density (OD) at 254 nm using an ISCO fractionator (Teledyne ISCO, Inc., Lincoln, NE, United States). RNA was extracted from each sucrose gradient fraction using TRIzol (Life Technologies). Reverse transcription was performed using a SuperScript III Reverse-Transcriptase Kit (Life Technologies) and random hexamers (Life Technologies) according to the manufacturer’s instructions. qRT-PCRs were carried out in a LightCycler 480 system using iQ Sybr Green Supermix (Bio-Rad) according to the manufacturer’s instructions using the following primers (*Bdnf-201* forward 5′-GCTTTGCGGATATTGCGAAGGGTT-3′, *Bdnf-201* reverse 5′-TGGAACATTGTGGCTTTGCTGTCC-3′, *ActB* forward*-5*′ -TGTGATGGTGGGAATGGGTCAGAA-3′, *ActB* reverse 5′-TGTGGTGCCAGATCTTCTCCATGT-3′). Results are presented in arbitrary units as relative amounts using serial dilutions of DRG RNA as qRT-PCR concentration standards. Experiments using this method of qRT-PCR (**Figure 2D**) were performed at McGill University.

### Statistics

**FIGURE 3:**
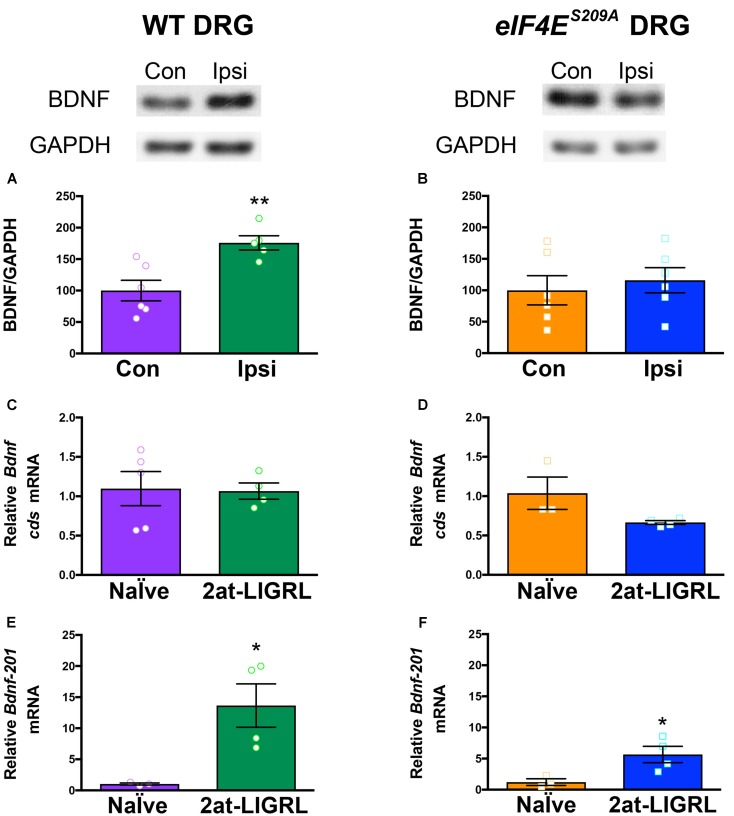
BDNF protein is increased in WT but not *eIF4E^S209A^* DRGs after PAR2 activation. **(A)** Western blot analysis shows an increase in BDNF protein standardized to GAPDH in WT DRGs (L4-L6) ipsilateral (IPSI) to 2at-LIGRL intraplantar (i.pl.) injection compared to contralateral (CON) (*n* ≥ 5, *t* = 3.662, *df* = 9, ^∗∗^*p* = 0.0052, *t*-test). Protein and mRNA extraction was done 24 h following i.pl. injection. **(B)** *eIF4E^S209A^* DRG (L4-L6) showed no differences in BDNF protein levels between IPSI and CON (*n* = 6, *p* > 0.05, *t*-test). mRNA levels of *Bdnf-*pan and the *Bdnf-201* isoform were analyzed using qPCR. **(C,D)** While *Bdnf-*pan mRNA levels after 2at-LIGRL injection were not changed in DRGs from both genotypes (*n* ≥ 3, *t*-test). **(E,F)** *Bdnf-201* mRNA levels were significantly increased in both WT and *eIF4E^S209A^* DRGs (WT: *n* ≥ 3, *t* = 3.055, *df* = 5, ^∗^*p* = 0.0283, *t*-test; *eIF4E^S209A^*: *n* ≥ 3, *t* = 2.756, *df* = 5, ^∗^*p* = 0.04, *t*-test).

**FIGURE 4:**
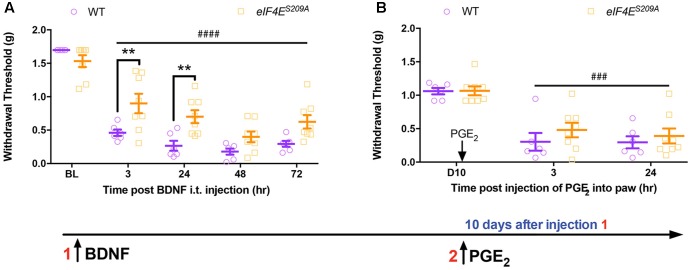
Administration of intrathecal BDNF evokes hyperalgesic priming in *eIF4E^S209A^* mice. **(A)** Intrathecal injection of BDNF (0.1 ng) was administered to both WT and *eIF4E^S209A^* mice. Acute mechanical hypersensitivity was blunted in *eIF4E^S209A^* mice at 3 and 24 h, but was equal in both genotypes by 48 h [*n* ≥ 6, *F*(1,60) = 18.97, *p* < 0.0001; *post hoc* Bonferroni’s ^∗∗^*p* = 0.0062, 0.0067, two-way ANOVA comparing WT vs. *eIF4E^S209A^* mice; ^####^*p* < 0.0001, two-way ANOVA compared to BL]. **(B)** PGE_2_ was injected i.pl. in WT and *eIF4E^S209A^* mice and equal hyperalgesic priming was observed in both genotypes (*n* ≥ 6, *p* > 0.05, two-way ANOVA comparing WT vs. *eIF4E^S209A^* mice; ^###^*p* = 0.0001, two-way ANOVA compared to BL).

All data are displayed as mean ± SEM, with individual samples represented within graphs to depict the *n* of each group and distribution. **Figures 1B,C**, **3A,B** represent band intensities normalized to GAPDH. **Figures 1D–H**, **3C–F** represent gene expression of 2^-ΔΔ*C*_T_^ normalized to *Gapdh*, then to WT samples. **Figure 2** displays mRNA relative amounts across sucrose gradient fractions. **Figures 4A,B** represents hindpaw withdrawal thresholds of each animal. GraphPad Prism 6 v 6.0 for Mac OS X was used for analysis. Statistical tests, *post hoc* analyses, and values for each figure are displayed in **Table 1**.

**Table 1:** Statistical tests used and values.

Test (Factor)	*F*(*df*1,*df*2) interaction *F*(*df*1,*df*2) row *F*(*df*1,*df*2) column	Corrected *t*-value, *df*	*p*-value	Adjusted *p*-value (*Post hoc* comparison)
Welch’s *t*-test unpaired (**Figure 1B**)	N/A	*t* = 2.106, Welch’s *df* = 18.51	*p* = 0.0491	N/A
Welch’s *t*-test unpaired (**Figure 1C**)	N/A	*t* = 0.01731, *df* = 5.056	*p* = 0.9869	N/A
Welch’s *t*-test unpaired (**Figure 1D**)	N/A	*t* = 1.205, Welch’s *df* = 5.681	*p* = 0.2762	N/A
Welch’s *t*-test unpaired (**Figure 1E**)	N/A	*t* = 0.1207, Welch’s *df* = 1.738	*p* = 0.9164	N/A
Welch’s *t*-test unpaired (**Figure 1H**)	N/A	*t* = 0.3394, Welch’s *df* = 3.257	*p* = 0.7550	N/A
Two-way ANOVA (**Figure 2B**)	*F*_i(13,56)_ = 12.5	N/A	*P*_i_< 0.001	F9: ^∗∗∗∗^*p* < 0.0001
	*F*_r(13,56)_ = 10.68		*P*_r_< 0.001	F12: ^∗∗∗∗^*p* < 0.0001
	*F*_c(1,56)_ = 1.192e^-006^		*P*_c_ = 0.9991	F13: ^∗∗∗∗^*p* < 0.0001
One-way ANOVA (**Figure 2C**)	*F*_(3,8)_ = 28.78	N/A	*p* = 0.0001	WT heavy vs. WT light: ^∗∗∗^*p* = 0.002
				WT heavy vs. *eIF4E*^S209A^ heavy: ^∗∗^*p* = 0.0011
Welch’s *t*-test unpaired (**Figure 2D**)	N/A	*t* = 0.2047, Welch’s *df* = 3.352	*p* = 0.8496	N/A
Welch’s *t*-test unpaired (**Figure 3A**)	N/A	*t* = 3.821, Welch’s *df* = 8.537	*p* = 0.0045	N/A
Welch’s *t*-test unpaired (**Figure 3B**)	N/A	*t* = 0.521, Welch’s *df* = 9.775	*p* = 0.6140	N/A
Welch’s *t*-test unpaired (**Figure 3C**)	N/A	*t* = 0.133, Welch’s *df* = 5.657	*p* = 0.8988	N/A
Welch’s *t*-test unpaired (**Figure 3D**)	N/A	*t* = 1.792, Welch’s *df* = 2.097	*p* = 0.2116	N/A
Welch’s *t*-test unpaired (**Figure 3E**)	N/A	*t* = 3.613, Welch’s *df* = 3.013	*p* = 0.0362	N/A
Welch’s *t*-test unpaired (**Figure 3F**)	N/A	*t* = 3.137, Welch’s *df* = 3.966	*p* = 0.0354	N/A
Two-way ANOVA (**Figure 4A**)	*F*_i(4,60)_ = 3.744	N/A	*P*_i_ = 0.0087	3 h: ^∗∗^*p* = 0.0062
	*F*_r(4,60)_ = 66.78		*P*_r_ < 0.0001	24 h: ^∗∗^*p* = 0.0067
	*F*_c(1,60)_ = 18.97		*P*_c_ < 0.0001	WT:
				BL vs. 3: ####*p* < 0.0001
				BL vs. 24: ####*p* < 0.0001
				BL vs. 48: ####*p* < 0.0001
				BL vs. 72: ####*p* < 0.0001
				*eIF4E*^S209A^:
				BL vs. 3: ####*p* < 0.0001
				BL vs. 24: ####*p* < 0.0001
				BL vs. 48: ####*p* < 0.0001
				BL vs. 72: ####*p* < 0.0001
Two-way ANOVA genotype (**Figure 4B**)	*F*_i(2,36)_ = 0.3779	N/A	*P*_i_ = 0.688	WT:
	*F*_r(2,36)_ = 32.6		*P*_r_ < 0.0001	D10 vs. 3: ####*p* < 0.0001
	*F*_c(1,36)_ = 1.291		*P*_c_ = 0.2634	D10 vs. 24: ####*p* < 0.0001
				*eIF4E*^S209A^:
				D10 vs. 3: ###*p* = 0.0001
				D10 vs. 24: ####*p* < 0.0001
Welch’s *t*-test unpaired (**Supplementary Figure S3**)	N/A	*t* = 2.146, Welch’s *df* = 3.821	*p* = 0.1017	N/A


## Results

### BDNF Protein Expression Is Decreased in the DRGs of eIF4E^S209A^ Mice

To test our hypothesis that *Bdnf* mRNA translation is regulated by eIF4E phosphorylation, we first determined the specificity of antibodies for immunodetection of BDNF protein in DRGs isolated from WT and *Bdnf*^+/-^ mice (Ernfors et al., 1994). As expected, DRGs isolated from *Bdnf*^+/-^ mice exhibited a markedly reduced expression of BDNF compared to WT DRGs (**Figure 1A**). As an additional control, we used liver, which expresses low levels of *Bdnf* mRNA (Yue et al., 2014). Protein levels of BDNF in liver lysates were low compared to DRGs (**Figure 1A**). The BDNF band was detected above the 20 kDa marker according to the predicted molecular weight (**Supplementary Figure S1A**, BDNF Ensembl, BDNF Uniprot). The same BDNF band observed in **Figure 1** was used for analysis throughout this study (**Supplementary Figure S1B**). After testing antibody specificity, we then measured BDNF protein in lysates from lumbar DRGs taken from both WT and *eIF4E^S209A^* mice (**Figure 1B**). We observed a significant decrease in BDNF protein expression in *eIF4E^S209A^* mice compared to WT. On the other hand, the levels of the BDNF receptor, trkB, were unchanged in lysates from lumbar spinal dorsal horn between genotypes (**Figure 1C**). A possible explanation for this deficit in BDNF protein is decreased *Bdnf* mRNA transcription in *eIF4E^S209A^* mice. To examine this possibility, we measured *Bdnf* mRNA levels using primers that recognize all *Bdnf* transcript variants by qPCR. In lumbar DRGs (**Figure 1D**) and lumbar spinal dorsal horn (**Figure 1E**), no differences were observed in *Bdnf cds* transcript abundance between genotypes. Additionally, there were no differences in *Gapdh* transcript abundance (**Supplementary Figure S2**). Mature *Bdnf* mRNA can assume a variety of different variants depending on 5′ UTR exon expression. At least 11 different 5′ UTR variants for *Bdnf* have been annotated and they are all encoded by different exons of varying lengths (Chiaruttini et al., 2008; Mele et al., 2015). In an effort to gain insight into why *Bdnf* mRNA translation is decreased in the absence of eIF4E phosphorylation, we plotted the transcript length vs. the Gibbs Free Energy (ΔG) of predicted 5′ UTR folds using the RNA structure prediction algorithm mfold^1^ (Mathews et al., 1999). We found that the transcript variant that is most strongly induced by pronociceptive factors in DRG, which contains the 5′ UTR encoded by the *Bdnf-201* isoform (Kim et al., 2001; Matsuoka et al., 2007; Obata et al., 2011; Salerno et al., 2012; Morioka et al., 2013; Uchida et al., 2013), is the longest and has the lowest ΔG score (**Figure 1F**). The predicted structure of the *Bdnf-201* isoform 5′ UTR is shown in **Figure 1G**. We therefore measured the abundance of this transcript variant in DRG using primers specific for the 5′ UTR encoded by *Bdnf-201* isoform. Again, we did not find any change in abundance of this transcript in lumbar DRGs from naïve mice of both genotypes (**Figure 1H**).

To directly evaluate whether eIF4E phosphorylation regulates *Bdnf-201* mRNA translation efficiency in DRG neurons, we sedimented and profiled polysomes isolated from lumbar and thoracic DRGs isolated from both genotypes. To obtain sufficient sample to conduct these experiments, we pooled DRGs from 5 mice per genotype. Extracts from DRGs of *eIF4E^S209A^* and WT mice were fractionated on sucrose density gradients (**Figure 2A**), and the distribution of *Bdnf-201* mRNA across these gradients was determined by qRT-PCR analysis using primers recognizing *Bdnf-201* transcripts. *Bdnf-201* mRNA associated with lighter polysome fractions in *eIF4E^S209A^* mice, as compared to WT mice (**Figures 2B,C**). Total *Bdnf-201* mRNA in pooled samples was not different between the two genotypes (**Figure 2D**). As an additional control, *ActB* associated with heavy polysome fractions in both WT and *eIF4E^S209A^* DRG samples (**Figure 2E**). These results indicate that *Bdnf-201* mRNA translation is influenced by eIF4E phosphorylation in DRGs.

### Activity-Dependent Bdnf mRNA Translation Is Regulated by eIF4E Phosphorylation

We have previously shown that protease-activated receptor 2 (PAR2)-induced hyperalgesic priming is dependent on BDNF signaling (Tillu et al., 2015) and is strongly reduced in *eIF4E^S209A^* compared to WT mice (Moy et al., 2017). Moreover, PAR2 activation promotes increased BDNF protein and mRNA transcript abundance in DRG neurons (Bao et al., 2014). We therefore sought to determine whether PAR2-induced changes in *Bdnf* gene expression are altered in *eIF4E^S209A^* mice. In WT mice, we observed that the PAR2-specific agonist 2at-LIGRL induced an increase in BDNF protein levels 24 h after hindpaw injection (**Figure 3A**). In contrast, PAR2 stimulation in *eIF4E^S209A^* mice failed to induce an increase in BDNF protein in affected DRGs (**Figure 3B**). The relative abundance of total *Bdnf* transcripts was not altered in DRG following PAR2 stimulation in the hindpaw in WT (**Figure 3C**) or *eIF4E^S209A^* (**Figure 3D**) mice. However, when we specifically measured changes in the *Bdnf-201* isoform abundance, we found a large increase in both WT (**Figure 3E**) and *eIF4E^S209A^* (**Figure 3F**) mice. No differences were observed between PAR2-induced *Bdnf-201* mRNA expression between WT and *eIF4E^S209A^* DRGs (**Supplementary Figure S3**). Therefore, while PAR2-induced enhancement of *Bdnf-201* transcription is eIF4E phosphorylation independent, eIF4E phosphorylation is required for this change in transcription to result in enhanced *Bdnf-201* mRNA translation.

### Intrathecal BDNF Induces Hyperalgesic Priming in eIF4E^S209A^ Mice

Based on these results, we predicted that direct injection of BDNF into the CNS should bypass any deficit in *Bdnf-201* mRNA translation in *eIF4E^S209A^* mice and produce full expression of hyperalgesic priming. BDNF (0.1 ng) was injected intrathecally in WT and *eIF4E^S209A^* mice and mechanical hypersensitivity was measured over the ensuing 72 h. While both genotypes displayed a significant drop in withdrawal threshold, early mechanical hypersensitivity magnitudes were decreased in *eIF4E^S209A^* mice compared to WT in response to BDNF (**Figure 1A**). On the other hand, no differences between genotypes were observed at 48 and 72 h after injection (**Figure 4A**). When mice were later challenged with PGE_2_ injection into the hindpaw, full hyperalgesic priming was clearly present in both genotypes (**Figure 4B**) despite the deficit in acute sensitization at early time points in *eIF4E^S209A^* mice. This is in marked contrast to observations in *eIF4E^S209A^* mice which show little, if any hyperalgesic priming in response to PAR2 agonist, nerve growth factor (NGF), interleukin 6 (IL-6) or carrageenan injection into the hindpaw (Moy et al., 2017). Hence, the deficit in hyperalgesic priming phenotype of *eIF4E^S209A^* mice can be rescued by direct injection of BDNF into the spinal cord.

## Discussion

**FIGURE 5:**
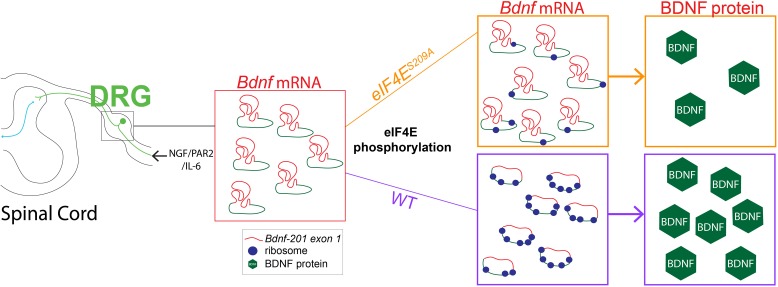
Linking decreased *Bdnf* mRNA translation to deficits in MNK1/2-eIF4E-regulated pain plasticity. Our results show that while NGF, IL-6, and PAR2 signaling require MNK1/2-eIF4E signaling to promote acute pain plasticity and the development of hyperalgesic priming. The development of hyperalgesic priming is mediated via a dependence on MNK1/2-eIF4E signaling for control of BDNF protein synthesis. In the absence of eIF4E phosphorylation enhanced *Bdnf-201* transcription proceeds but this fails to lead to enhanced translation of BDNF protein, likely because eIF4E phosphorylation is required to efficiently translate this mRNA species that has a highly structured 5′ UTR (shown in red, coding sequence in blue). We propose that this failure to enhance *Bdnf-201* mRNA translation leads to a deficit in hyperalgesic priming in *eIF4E^S209A^* mice.

We have identified a novel *bona fide* eIF4E phosphorylation target mRNA in the nervous system: *Bdnf*. Our results show that *Bdnf-201* mRNA levels are normal in *eIF4E^S209A^* mice, but BDNF protein levels are profoundly decreased and PAR2 activation-induced changes in *Bdnf* mRNA translation fail to materialize in the absence of eIF4E phosphorylation despite a robust increase in the transcriptional response (**Figure 5**). Because BDNF plays a core function in plasticity at the first synapse in the pain pathway (Pezet and McMahon, 2006) and in many other brain circuits (Lu et al., 2008), it is curious that brain plasticity phenotypes in these mice are not readily observed (Gkogkas et al., 2013, 2014). A possible explanation for this comes from the 11 exons capable of encoding the 5′ UTR of the *Bdnf* mRNA. The primary exon induced by injury or pronociceptive factors in the DRG is the *Bdnf-201* isoform, which encodes the longest 5′ UTR with the most extensive predicted secondary structure. In brain, the primary 5′ UTR exon in mice is exon 4, which is much shorter and contains a less complex secondary structure (Chiaruttini et al., 2008; Mele et al., 2015). Hence, eIF4E phosphorylation may preferentially influence efficient translation of BDNF transcripts, such as those found in DRG, where exon choice contributes to long 5′ UTRs with extensive secondary structure (Matsuoka et al., 2007). Another component of the eIF4F complex, eIF4A, influences the translation of mRNAs with long, highly structured 5′ UTRs (Parsyan et al., 2011). Because eIF4A is an RNA helicase, this likely reflects unwinding of secondary structures leading to more efficient translation. Recent studies have demonstrated that eIF4E enhances eIF4A activity (Feoktistova et al., 2013) but the role of eIF4E phosphorylation in this process is unknown.

It is unlikely that all of the behavioral phenotypes displayed by *eIF4E^S209A^* mice (Moy et al., 2017) are explained by inefficient *Bdnf* mRNA translation. BDNF is released by DRG neurons in an activity-dependent fashion and influences spinal cord excitability in response to nociceptor activation (Zhou et al., 2008; Bao et al., 2014; Chen et al., 2014). BDNF plays a key role in the generation of pain plasticity, including plasticity in hyperalgesic priming models (Melemedjian et al., 2013). However, a key feature observed in *eIF4E^S209A^* mice is a deficit in enhanced intrinsic excitability in nociceptors in response to NGF, IL6, or PAR2 activation (Moy et al., 2017). This lack of hyperexcitability is likely the reason explaining why *eIF4E^S209A^* mice display reduced mechanical hypersensitivity at the early time points post intrathecal BDNF injection compared to WT mice. BDNF can activate presynaptic trkB receptors on the DRG (Lin et al., 2011), inducing hypersensitivity in WT mice, whereas in *eIF4E^S209A^* mice, nociceptor hyperexcitability is diminished creating a blunted effect early on. This change in nociceptor excitability is likely driven by the activity-dependent translation of proteins that alter ion channel trafficking, or the increased translation of ion channels themselves. These mRNAs have yet to be identified. Our findings point to multiple points at which translation regulation influences excitability in the pain system through altered gene expression in nociceptors. One of these is the translation of new proteins that alter the excitability of the neuron. The other is the increased translation of a key neuromodulatory protein that induces synaptic plasticity in the neurons of the spinal dorsal horn when it is released from DRG neurons. It is interesting that both of these steps appear to be regulated by the same signaling mechanism – MNK-mediated phosphorylation of eIF4E. This highlights the importance of this signaling axis as a potential therapeutic target for pain plasticity.

Our work identifies a novel target mRNA for eIF4E phosphorylation, *Bdnf-201*. Other mRNA targets for phosphorylated eIF4E include cytokines and chemokines discovered through polysome profiling in mouse embryonic fibroblasts and MMP2 and 9 which were identified in the CNS (Furic et al., 2010; Gkogkas et al., 2014). While eIF4E phosphorylation was discovered more than 2 decades ago as a novel mechanism of translation regulation, the precise mechanisms through which this signaling mechanism controls the translation of distinct mRNAs has yet to be elucidated. Our work is consistent with a model wherein long, highly structured 5′ UTRs are important for eIF4E phosphorylation-mediated translation control, but this hypothesis requires further examination. Discovering the full repertoire of phosphorylated eIF4E mRNA targets will have important implications for a variety of disease states associated with enhanced eIF4E phosphorylation.

## Author Contributions

JM and AK collected tissue for this study. JM prepared samples, ran, and analyzed western blotting and qRT-PCR. AK executed the polysome assay and analysis. MA performed the behavioral assay. JM and TP designed the original experiments and drafted the manuscript. All authors contributed to the interpretation of the data, intellectual content, edited, and approved the final manuscript.

## Conflict of Interest Statement

The authors declare that the research was conducted in the absence of any commercial or financial relationships that could be construed as a potential conflict of interest.

## Footnotes

1 http://unafold.rna.albany.edu/?q=mfold

## Supplementary Material

**FIGURE S1 |** BDNF migrates to approximately 29kDa. **(A)** Entire western blot indicating the size of each protein standard (20-250 kDa) with protein from 1) WT DRGs, 2) *Bdnf*^+/-^ DRGs, and 3) WT liver. BDNF protein was detected at above the 20 kDa marker. **(B)** Entire western blot containing 1) WT1 DRGs, 2) *eIF4E^S209A^*1 DRGs, 3) WT2 DRGs, 4) *eIF4E^S209A^*2 DRGs, 5) WT3 DRGs, 6) *eIF4E^S209A^*3 DRGs, 7) WT4 DRGs, 8) *eIF4E^S209A^*4 DRGs, 9) WT5 DRGs, 10) *eIF4E^S209A^*5 DRGs, 11) WT6 DRGs, 12) WT7 DRGs, and 13) *eIF4E^S209A^*6 DRGs. Green arrows indicate the predicted molecular weight (∼29kDa).

**FIGURE S2 |** Levels of *Gapdh* mRNAs are equal between WT and *eIF4E^S209A^* DRGs. Amplification plot of *Gapdh* mRNAs from both naïve WT and *eIF4E^S209A^* DRGs demonstrating no differences in *Gapdh* mRNA quantities.

**FIGURE S3 |** PAR2 induced *Bdnf-201* mRNA expression are not different between WT and *eIF4E^S209A^* DRGs. Relative expression of *Bdnf-201* in both WT and *eIF4E^S209A^* DRGs 24h post 2at-LIGRL injection exhibit no differences between genotypes.
