# Supplementary figures and images for: eIF4E Phosphorylation Influences Bdnf mRNA Translation in Mouse Dorsal Root Ganglion Neurons
Source: Front Cell Neurosci. 2018 Feb 6;12:29. doi: 10.3389/fncel.2018.00029 (PMC5808250; doi:10.3389/fncel.2018.00029)

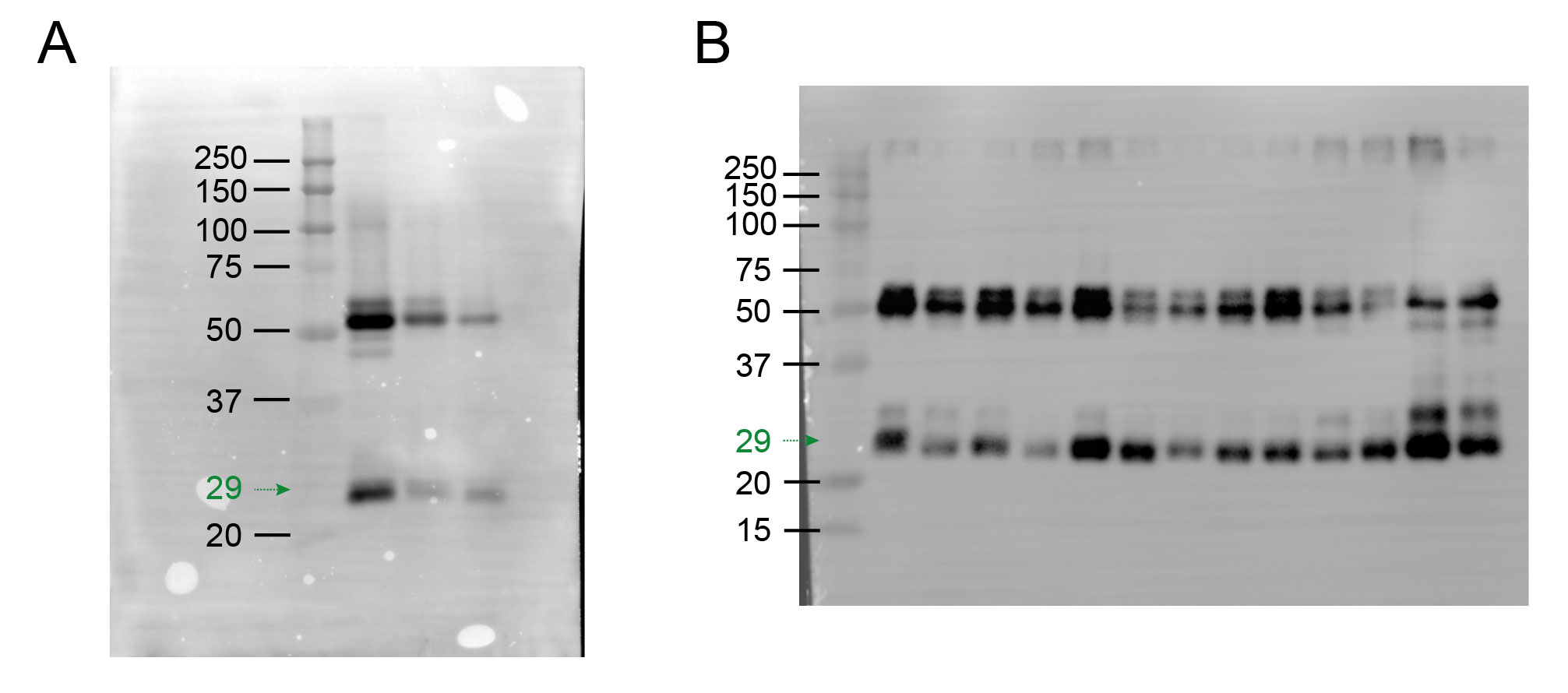

Supplement: Supplementary file 1 [file Image_1.JPEG]

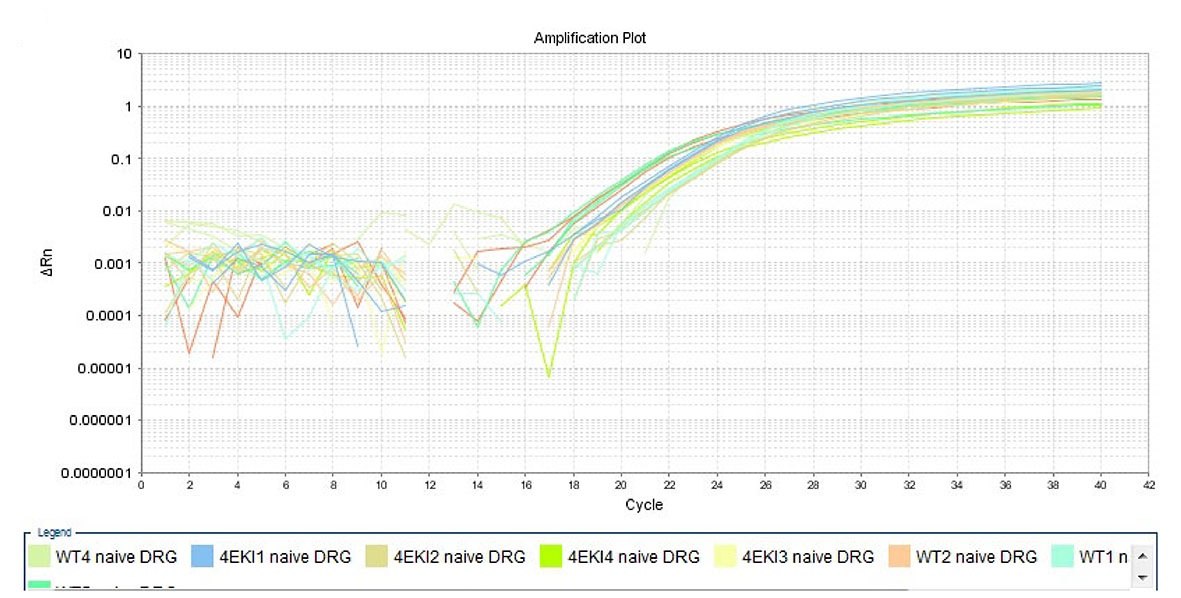

Supplement: Supplementary file 2 [file Image_2.jpg]

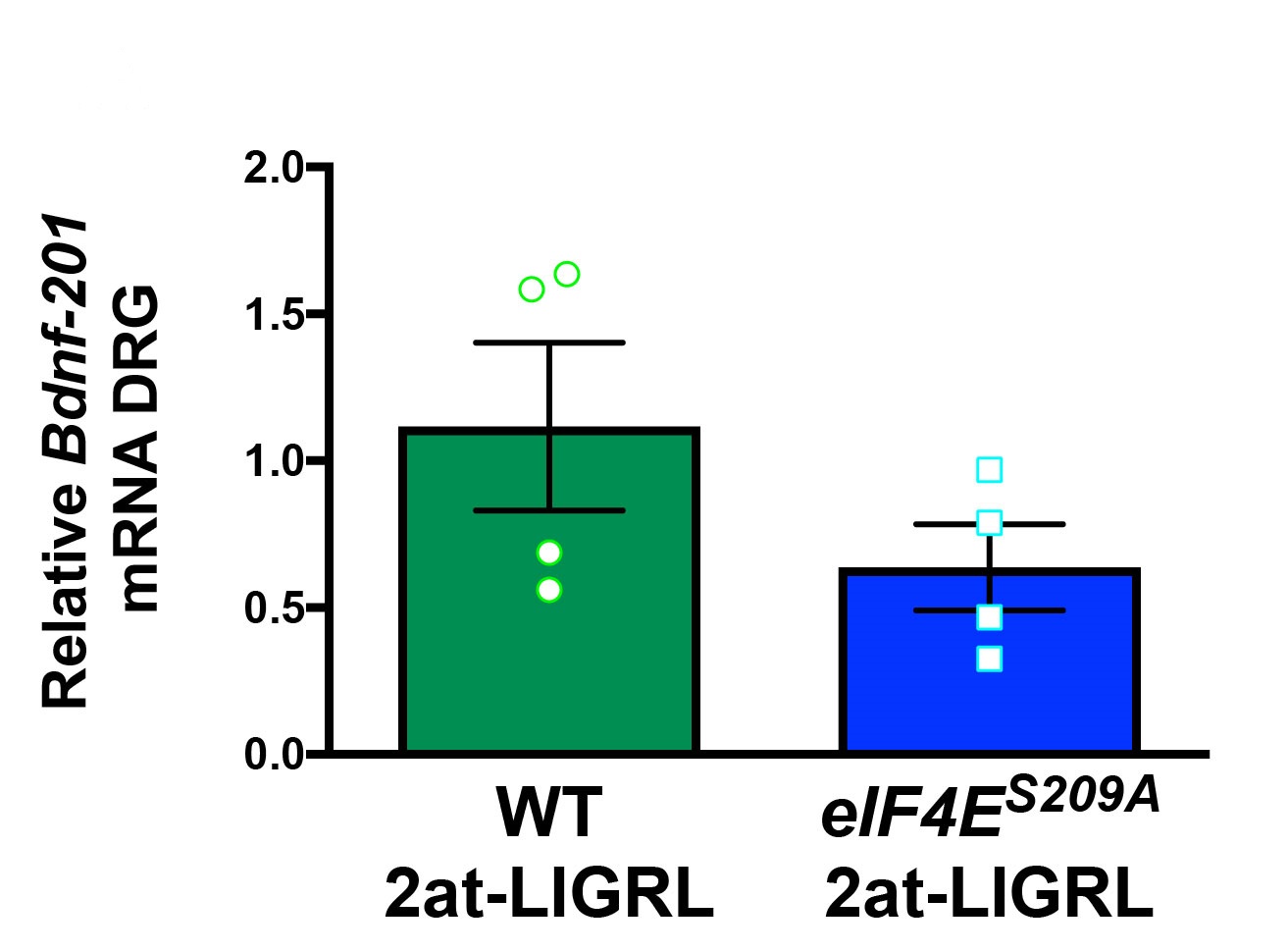

Supplement: Supplementary file 3 [file Image_3.jpg]
